# From Detection to Surveillance: The Evolving Role of Micro-Ultrasound in Prostate Cancer

**DOI:** 10.3390/diagnostics16152364

**Published:** 2026-07-28

**Authors:** Darius Ashrafi, Laurence Klotz

**Affiliations:** 1 Division of Urology, Sunnybrook Health Sciences Centre, University of Toronto, Toronto, ON M4N 3M5, Canada; d.r.ashrafi@gmail.com; 2 Surgical Outcomes Research Centre (SOuRCe), Royal Prince Alfred Hospital, Sydney, NSW 2050, Australia

**Keywords:** micro-ultrasound, prostate cancer, csPCa, targeted biopsy, active surveillance, artificial intelligence, mpMRI

## Abstract

High-resolution micro-ultrasound (microUS) is a 29 MHz imaging modality that allows visualisation of prostatic ductal architecture at 70 µm resolution, enabling real-time recognition of architectural disruption associated with clinically significant prostate cancer (csPCa). This review summarises the current evidence for microUS in the diagnosis and management of prostate cancer, with emphasis on developments since the pivotal OPTIMUM phase-3 randomised controlled trial. OPTIMUM provided level 1 evidence for microUS-guided biopsy as non-inferior to MRI-guided biopsy for csPCa detection. We examine the PRI-MUS risk identification protocol, the pre- and post-OPTIMUM evidence base, biopsy targeting and core number, inter-observer reliability and the learning curve, and emerging applications in active surveillance, restaging biopsy, local staging and tumour characterisation, and post-treatment surveillance for local recurrence. We also review the growing body of work on artificial intelligence-enhanced microUS, alongside workflow, cost, and environmental considerations. Whilst inter-reader variability and the learning curve remain barriers, microUS offers comparable sensitivity to multiparametric MRI with practical advantages in cost, accessibility, and workflow. In our view, microUS warrants acceptance as a primary imaging modality for the work-up of suspected prostate cancer.

## 1. Introduction

Prostate cancer incidence is projected to double by 2040, with the greatest increase in countries with a developing diagnostic health infrastructure. Accurate identification of clinically significant prostate cancer (csPCa—International Society of Urological Pathology (ISUP) grade group ≥ 2) is an important public health priority. It ensures recognition and treatment of aggressive disease while identifying and avoiding over-treatment of indolent disease [1].

Prostate biopsy has been performed since the early twentieth century with finger-guided transrectal needle biopsy. In the 1970s, transrectal ultrasonography (TRUS) was developed by urologists as an imaging modality for the prostate and first used for systematic prostate biopsies in the 1980s [2]. The conventional 6–12 MHz TRUS allows for reliable needle placement and gross zonal anatomy. However, it does not image the majority of prostate cancers. Thus, systematic ‘blind’ sampling remained the standard diagnostic approach for decades. The well-recognised sequelae of this have been over-detection of indolent disease and under-sampling of csPCa [2].

Multiparametric magnetic resonance imaging (mpMRI), standardised by the Prostate Imaging Reporting and Data System (PI-RADS), has reshaped the diagnostic pathway by providing pre-biopsy localisation and supporting targeted biopsy via cognitive or software-based MRI/TRUS fusion approaches [3]. The landmark PROMIS and PRECISION trials demonstrated the superiority of this approach. PROMIS demonstrated an mpMRI sensitivity of 93% vs. 48% and a negative predictive value of 89% vs. 74% compared to TRUS biopsy for csPCa. Overall, 27% of men could have safely avoided a biopsy based on a negative MRI [4]. PRECISION found that with MRI, significantly more csPCa (38% vs. 26%, *p* = 0.005) and fewer clinically insignificant cancers (9% vs. 22%, *p* < 0.001) were detected compared to the systematic TRUS biopsy [5]. As a result, most international guidelines recommend a pre-biopsy mpMRI as the standard of care for men with concerns of csPCa [6].

mpMRI, however, has well-documented limitations. There is substantial inter-reader variability in reporting, particularly for PI-RADS 3 lesions. False negative rates for csPCa are 10–15% [4,7,8]. MR image quality is degraded by the presence of hip arthroplasties [9]. From a patient’s perspective, it requires cooperation and gadolinium contrast, although the latter is being challenged by biparametric MRI [10]. There are several patients who are simply ineligible or unwilling due to pacemakers, severe claustrophobia or renal impairment. mpMRI is costly and resource intensive, requiring capital investment, scanner availability and MR technologist time, radiologist expertise, reporting and time [11]. This is a significant constraint in many health systems, including those in the ‘first world’ [11]. In Canada, MRI waiting times are often 3–6 months due to lack of accessibility [12].

High-resolution micro-ultrasound (microUS) was developed in the mid-2010s [13]. It is a point-of-care alternative to TRUS that can be used directly at the time of biopsy [13,14]. It is portable, small, and compatible with an outpatient urology clinic environment. It images at 70 µm resolution, allowing real-time visualisation of the architectural disruption associated with csPCa [13,14]. The pivotal OPTIMUM phase-3 RCT provided level 1 evidence for microUS-guided biopsy [15]. microUS is now established as a diagnostic tool and role in prostate cancer management is currently being further investigated [16].

This comprehensive review summarises the current evidence base for microUS in the diagnosis and management of prostate cancer, with attention to developments since the pivotal OPTIMUM trial. There are numerous recent publications covering systematic reviews [17], real-world data [18], screening [19], active surveillance [19], post-prostatectomy recurrence [20], AI-enhanced microUS [21], and economic and environmental applications [22]. We searched PubMed and Google Scholar for English-language articles published between January 2020 and June 2026. Search terms were combined and included ‘prostate cancer’, ‘micro-ultrasound’, ‘PRI-MUS’, ‘multiparametric MRI’, ‘active surveillance’, ‘local staging’, ‘biochemical recurrence’, and ‘artificial intelligence’. Randomised controlled trials, systematic reviews and prospective cohort studies were prioritised. Smaller retrospective series and feasibility studies were included where they were the only available literature in each domain. As this is not a systematic review, we did not undertake a systematic search, risk-of-bias assessment, or quantitative synthesis, and this review is not reported in accordance with PRISMA standards.

## 2. Micro-Ultrasound

Conventional TRUS images tissue alterations at a spatial scale larger than prostate cancer lesions; hypoechoic lesions are therefore neither sensitive nor specific for clinically significant prostate cancer [13]. Healthy peripheral zone prostatic ducts measure approximately 100 µm in diameter, and prostate cancer is histologically characterised by disordered glandular architecture [14].

The only commercially available microUS platform at the time of writing is the ExactVu system (Exact Imaging, Markham, ON, Canada). This device uses a 29 MHz side-fire transducer (EV29L) containing 512 individual piezoelectric crystals packed at 90 µm intervals [13]. The packing of crystals is approximately 3 times more dense than on a conventional ultrasound probe. This permits imaging to a distance of 6 cm, overcoming the more limited depth of penetration of higher frequency ultrasound and allowing the entire gland to be visualised in a majority of patients. The major microUS studies have not imposed an upper prostate volume limit [13]. At this resolution, microUS can assess ductal architecture and its disruption directly. It recognises peripheral and transition zone lesions, capsular extension, and prior needle tracks in real time during the biopsy procedure [14].

Since 2017, the ExactVu platform has incorporated MRI fusion software (FusionVu), enabling side-by-side or fused display of pre-acquired mpMRI with the live microUS sweep. Fusion is useful where mpMRI has already been performed but is not a prerequisite for microUS-guided targeting. Three clinically valid approaches exist: software-based MRI-microUS fusion, cognitive fusion using MRI images, and primary targeting based on microUS without prior MRI [15]. MicroUS can therefore function within an MRI-first pathway or independently where timely MRI is unavailable or contraindicated [23].

## 3. PRI-MUS: The Risk Identification Protocol

**Figure 1:**
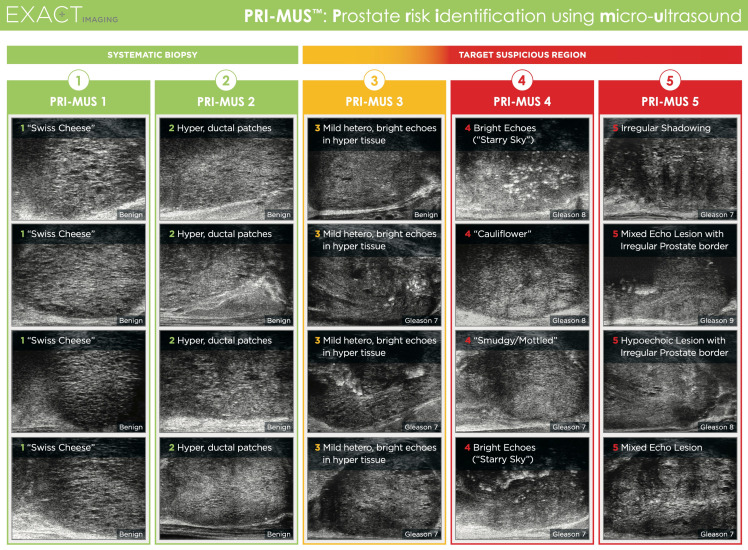
The PRI-MUS risk identification grading—The Prostate Risk Identification using Micro-UltraSound (PRI-MUS) five-point scale [24]. Image reproduced with permission from Exact Imaging, Markham, ON, Canada, 2026.

The Prostate Risk Identification using Micro-UltraSound (PRI-MUS) protocol was developed in 2016 with the use of cine loops from 400 biopsies in a multi-institutional randomised trial and subsequent validation with an independent pathology-blinded review of 100 cases (Figure 1) [14]. PRI-MUS is a five-point Likert risk scale, analogous to the PI-RADS [14]. PRI-MUS 1 represents normal anatomy whilst PRI-MUS 5 corresponds to the highest likelihood of csPCa [14]. An increasing PRI-MUS score correlates with an increasing rate of detection of grade group 2 prostate cancer and above [14]. PRI-MUS 3 is commonly used as a threshold for biopsy [14].

This original protocol was optimised for the peripheral zone [14]. Anterior and transitional zone lesions are more difficult to identify due to their heterogeneous architecture, peri-urethral calcifications and corpora amylacea with shadowing anteriorly, and signal attenuation with increasing distance from the probe [25]. As a result, the sensitivity of anterior lesions is reduced [25].

**Figure 2:**
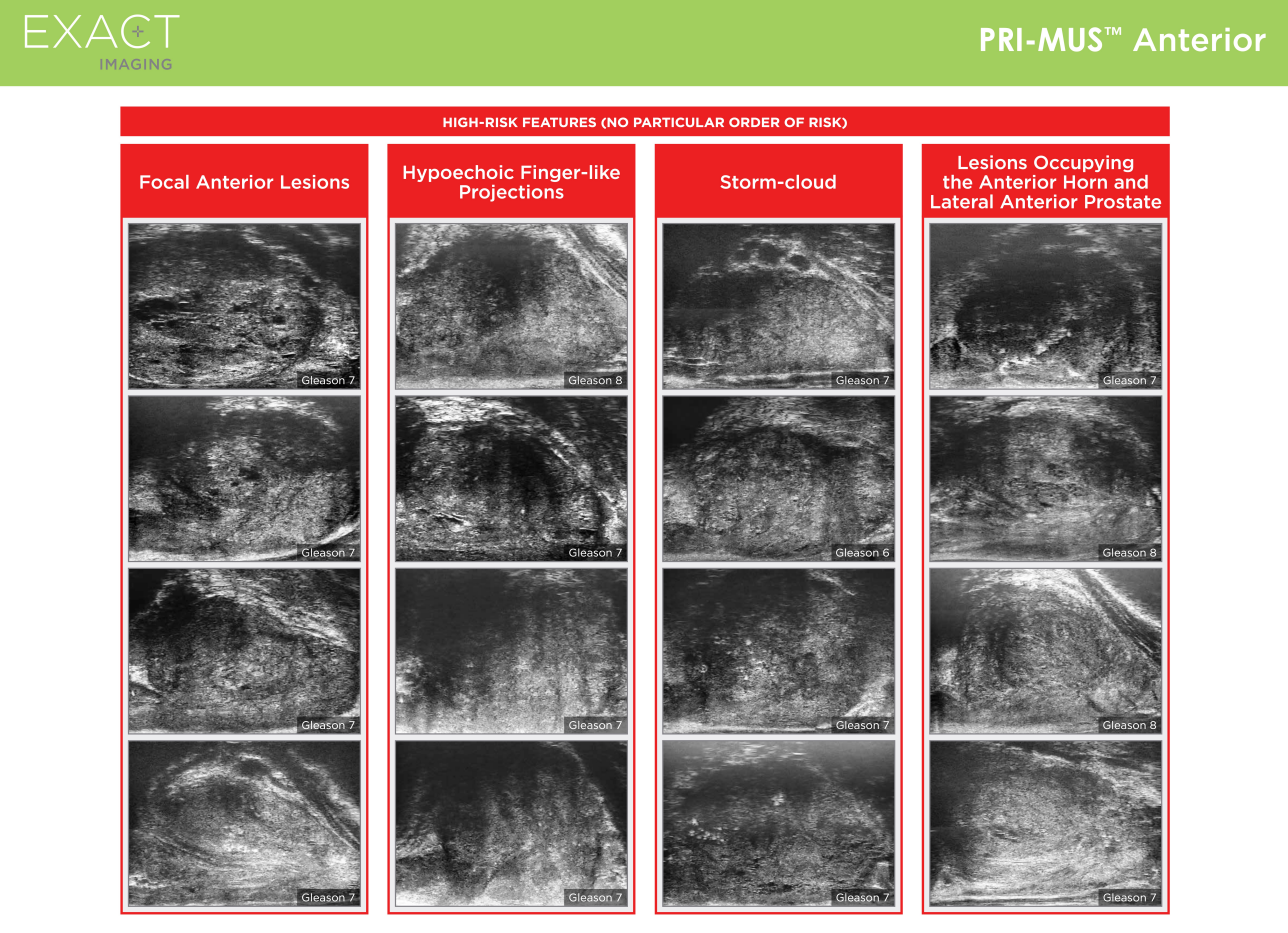
Anterior PRI-MUS grading describes high-risk features of anterior and transition zone lesions. These are examples of such features, including: focal anterior lesions, hypoechoic finger-like projections, the “storm-cloud” pattern, and lesions occupying the anterior horn and lateral anterior prostate. Note these are not presented in order of increasing risk, but simply as examples [26]. Image reproduced with permission from Exact Imaging, Markham, ON, Canada, 2026.

The Anterior PRI-MUS grading tool was developed and validated in 2023 to address this issue (Figure 2) [25]. A total of 102 select prostate scans were used from patients who underwent subsequent radical prostatectomy [25]. Urologists interpreted the images and categorised the likelihood of csPCa in the anterior zone as likely, equivocal and unlikely [25]. The diagnostic performance showed a median sensitivity of 72%, a specificity of 68%, and an AUC of 0.75 [25]. However, there was overall poor inter-reader agreement (Fleiss kappa 0.18) [25]. This protocol is applied together with the standard PRI-MUS score for whole-gland assessment [25].

In 2026, Corona and colleagues proposed an extension to the PRI-MUS framework with echogenicity as an adjunct feature [27]. They reviewed 229 prostate lesions from 181 men who underwent microUS-guided transperineal biopsies [27]. The lesions were visually graded as hyperechoic, isoechoic, or hypoechoic relative to the central zone, which acted as an internal reference, due to its consistent tissue characteristics and low malignancy risk [27]. Decreasing echogenicity was an independent predictor of prostate cancer grade. The PPV for csPCa with hyperechoic lesions was 22%, compared to 56% for isoechoic and 62% for hypoechoic lesions (*p* < 0.05) [27]. Hyperechoic lesions contained a higher proportion of benign tissue compared to isoechoic and hypoechoic lesions [27]. More significant cancers (e.g., grade groups 4 and 5) were absent from hyperechoic lesions [27]. Echogenicity also correlated with the PRI-MUS score amongst those with csPCa [27]. Hypoechoic lesions were present in 13% of PRI-MUS 3, 35% of PRI-MUS 4, and 86% of PRI-MUS 5 lesions [27]. The findings correlate with a significant decrease in apparent diffusion coefficient values on co-registered MRIs [27]. The proposed refinement to PRI-MUS scoring reclassifies hyperechoic lesions as PRI-MUS 3 and restricts PRI-MUS 4 and 5 to isoechoic and hypoechoic lesions [27].

## 4. The Evidence Base

### 4.1. Pre-OPTIMUM Landscape

Early prospective work focused on the diagnostic accuracy of microUS against mpMRI in suspected prostate cancer with several prospective and retrospective studies (Table 1). Several prospective trials have reported microUS sensitivity to range between 90 and 94% and negative predictive value between 82 and 85% [28,29]. Another prospective cohort study with 203 patients found microUS to be non-inferior to MRI and TRUS fusion for grade group 2 and above [30]. In a retrospective review of men with equivocal PI-RADS 3 lesions, blinded microUS retained high sensitivity and NPV but low specificity, supporting a role as a risk-stratifier [31].

In biopsy-naïve men with negative MRIs, microUS detected more csPCa than standard biopsy, and additional cancers were identified amongst those with PRI-MUS 3 or greater [32,33]. PRI-MUS 3 and greater, with a PSA density greater than 0.15, was also predictive for csPCa [32,33]. When microUS was also negative (in approximately 22%), avoiding a biopsy would only have missed one csPCa [33]. Logistic regression models identified positive microUS, age, digital rectal examination, and PSA density greater than 0.15 as independent predictors of prostate cancer [33].

The relatively low specificity of PRI-MUS ≥ 3 was an early limitation of the technology. In retrospect, this appears to reflect an early learning curve experience. More recent data from the OPTIMUM trial discussed below indicates that with greater operator experience, specificity increases.

**Table 1:** Studies comparing the diagnostic performance of microUS in the detection of csPCa.

Author/Trial (Year)	*n*	Design	Comparison and Findings	Ref.
***Pre-OPTIMUM diagnostic studies***				
Klotz (2021)	1040	Prospective multicentre registry	microUS vs. mpMRI, head-to-head in the same men: microUS sensitivity 94%, NPV 85% for csPCa	[28]
Lughezzani (2021)	320	Prospective, single-institution	microUS, read blinded to MRI, vs. the prior MRI before fusion biopsy: microUS sensitivity 90%, NPV 82%	[29]
Avolio (2021)	111	Single-institution, equivocal lesions	microUS vs. biopsy histology in PI-RADS 3 lesions: sensitivity 100%, NPV 100%; specificity 34%, PPV 27%	[31]
Hofbauer (2022)	203	Prospective non-inferiority	microUS (blinded to MRI) vs. MRI-TRUS fusion biopsy in the same session: microUS non-inferior for GG ≥ 2 detection	[30]
Avolio (2023)	125	Prospective, negative MRI	microUS-targeted plus systematic biopsy vs. histology in biopsy-naïve men with negative MRI: sensitivity 97%, NPV 96%; specificity 30%, PPV 34%	[33]
Albers (2024)	103	Retrospective, negative MRI	microUS-guided vs. standard systematic biopsy in biopsy-naïve men with negative MRI: csPCa 25% vs. 17%; PRI-MUS ≥ 3 with PSAD ≥ 0.15 predictive	[32]
***Pivotal randomised controlled trial***				
Kinnaird—OPTIMUM (2025)	678	Phase 3 RCT, 20 centres/eight countries	microUS-guided vs. mpMRI/microUS fusion vs. mpMRI/TRUS fusion (standard of care): both microUS arms non-inferior to standard of care for csPCa (10% margin)	[15]
***Post-OPTIMUM studies***				
Castilho Borges (2026)	1119 (767/352)	Retrospective, propensity-weighted	microUS-guided biopsy vs. MRI/US fusion biopsy in men with MRI-visible lesions: csPCa 45% vs. 34% (combined) and 39% vs. 24% (systematic); no difference for targeted cores alone; less upgrading at radical prostatectomy with microUS, 17% vs. 38%	[34]
Sanz Gomez (2026)	200	Prospective real-world, transperineal	Real-world microUS/PRI-MUS biopsy vs. radical prostatectomy histology: PCa 62%, csPCa 42%; at PRI-MUS ≥ 4 sensitivity 93%, NPV 83%; ISUP grade concordance with RP 85%; csPCa in 17% of 30 men with negative/absent mpMRI	[35]
Forrest (2026)	169	Retrospective, mpMRI-first pathway	mpMRI-informed microUS vs. historical mpMRI/US fusion data: 84% (206/244 lesions) of MRI lesions visible on microUS; csPCa 41% rising to 43% with microUS-only lesions; PPV for csPCa 0.37 if a microUS correlate was seen vs. 0.18 if not (*p* < 0.05)	[36]
***Meta-analyses (2026)***				
Lu (2026)	3667 (microUS) 3887 (mpMRI)	11 prospective studies (one registry RCT)	microUS- vs. mpMRI-guided biopsy: no difference in csPCa detection (OR 1.13, 0.99–1.30; *p* = 0.07)	[37]
Micheleto (2026)	5581 (microUS) 5937 (mpMRI)	22 studies (observational + RCT)	microUS- vs. MRI-targeted biopsy: comparable csPCa detection, pooled detection ratio 1.04 (0.95–1.15); 2037 vs. 2043 csPCa cases	[38]
Garcia-Becerra (2026)	2626	Eight prospective studies (1 RCT)	microUS vs. mpMRI, pooled: sensitivity 0.87 vs. 0.88 (NS, *p* = 0.72); specificity 0.25 vs. 0.30 (significantly favouring mpMRI); overlapping summary ROC	[17]
Abdel Gawad (2026)	3640	Standalone microUS, no mpMRI comparator	microUS alone vs. biopsy histology, PRI-MUS ≥ 3: pooled sensitivity 0.84, specificity 0.41; DOR 3.95	[39]

### 4.2. OPTIMUM: Level 1 Evidence

The OPTIMUM trial was the first phase 3, prospective, open-label non-inferiority RCT comparing microUS to mpMRI (Table 1) [15]. A total of 802 biopsy-naïve men from 20 centres in eight countries were recruited with a 1:2:3 randomisation between microUS-guided, mpMRI and microUS fusion, and mpMRI and conventional TRUS fusion (standard of care) between December 2021 and September 2024. The trial originally had a planned cohort of 1200, with an interim analysis planned at 600 patients, and a stopping rule should non-inferiority be demonstrated at that point. In fact, that is what occurred. A total of 678 patients were randomised and evaluable. These men had a targeted biopsy and synchronous systematic biopsy regardless of imaging findings. The rates of detecting csPCa were similar between all three arms. With a prespecified non-inferiority margin of 10%, both the microUS and mpMRI/microUS arms were non-inferior to the standard of care (SOC) of mpMRI/TRUS fusion in detecting csPCa. When reviewing targeted-only detection rates, these groups were also non-inferior to the SOC [15].

### 4.3. New Evidence Since the OPTIMUM Trial

The post-OPTIMUM evidence addresses three areas: comparative performance against mpMRI, real-world standalone performance and its role as a complementary tool in an mpMRI-first diagnostic pathway (Table 1).

A propensity-weighted analysis of 1119 men with MRI-visible lesions found that microUS-guided biopsy detected significantly more csPCa than MRI/TRUS fusion and was associated with significantly less pathological upgrading at the time of radical prostatectomy [34]. In the study, there were no differences when reviewing target biopsies alone.

Four meta-analyses published to date in 2026 explored the comparative performance of microUS in prostate cancer detection rates (Table 1). For those that compared microUS to mpMRI, sensitivity for csPCa was equivalent and detection rates did not differ [17,37,38]. One pooled analysis did demonstrate lower specificity for microUS; however, the summary ROC curves overlapped for both modalities [17]. One meta-analysis assessed studies with microUS as a standalone test with comparison to histopathology, demonstrating that PRI-MUS 3 and above lesions had a pooled sensitivity of 0.84 and specificity of 0.41 [39].

Across the meta-analyses, the consistent finding was that microUS demonstrates comparable high sensitivity to mpMRI in the detection of csPCa with an associated high negative predictive value. Particularly when combined with PSA density, it supports its use as a rule-out test.

Real-world data supports these findings. Sanz-Gomez et al. reported the largest series to date, with rising csPCa detection with rising PRI-MUS score and high concordance with radical prostatectomy histopathology [35]. For PRI-MUS 4 and above, the csPCa sensitivity was 93%. ISUP grade concordance with radical prostatectomy was 85%, with 9% upgraded and 6% downgraded. This compares favourably to historical concordance rates with TRUS only of 55–68%, MRI-targeted of 69.5–76% and other microUS cohorts of 73–84% [39,40,41,42].

The complementary role of microUS within an mpMRI-first pathway was explored in a single-centre retrospective study of 169 consecutive men who underwent an mpMRI, followed by an mpMRI-informed microUS-guided biopsy between 2020 and 2023 [36]. They compared detection rates of prostate cancer to their institutional data of mpMRI/TRUS. microUS was performed blinded to mpMRI. Of the 244 lesions identified on mpMRI, 206 (84%) were prospectively visible on microUS. These could be targeted in real time with cognitive fusion only. Higher PI-RADS lesions were more likely to be visible on microUS. Overall, 10% of lesions identified by microUS were not visible on mpMRI and resulted in an additional three csPCa cases, increasing csPCa detection from 41% to 43%. The PPV remained unchanged with the addition of microUS compared to mpMRI/TRUS fusion [36].

## 5. Biopsy Targeting, Approach, and Core Number

mpMRI-TRUS cognitive and software fusion carries intrinsic registration error due to prostate motion between mpMRI and biopsy, prostate deformation by transrectal probe, software inaccuracies and cognitive operative variability. MRI fusion studies have demonstrated that two to five cores are needed to compensate for registration error [43,44]. microUS removes that registration error by visualising the lesion and the needle trajectory through the lesion in real time. A microUS single-centre series showed that a three-core targeted biopsy increased csPCa detection rates [45]. The OPTIMUM trial mandated three targeted cores per PRI-MUS 3–5 lesion [15].

Black et al. compared office-based transperineal microUS biopsy with software fusion mpMRI/TRUS [23] in a retrospective single-centre review of 698 men between 2019 and 2023. The transperineal arm included 306 patients compared to 392 patients in the transrectal arm. In biopsy-naïve men, transperineal microUS demonstrated significantly higher csPCa detection (53% vs. 42%, *p* = 0.01). The transperineal microUS approach also required significantly fewer cores for this superior detection rate (11 ± 5 vs. 15 ± 4 cores, *p* < 0.01). Upgrading from grade group 1 to grade group 2 or higher was less common after microUS. No patient in the transperineal arm developed urosepsis, compared with two in the transrectal arm. The limitation was the retrospective, non-randomised design and comparison of transperineal and transrectal techniques with microUS and mpMRI/TRUS. However, real-time adjustment with microUS reduces registration and sampling errors. The transperineal approach also aligns with several studies showing it lowers post-biopsy infection risk [46].

## 6. Inter-Observer Reliability and the Learning Curve

MicroUS requires real-time interpretation by the operator; therefore, variability in interpretation has direct procedural consequences. The operator’s ability and confidence in identifying, interpreting and targeting lesions affects the accuracy of targeting and cancer detection. Zhou et al. conducted a multi-reader variability study using digital volumetric annotation to assess agreement amongst microUS interpreters of varying experience levels [47]. Reader experience with microUS ranged from two to six years and was not strongly correlated with agreement. The mean reader sensitivity was 0.66; however, it rose to 0.87 when anterior lesions were excluded. Reassuringly, grade group 3 and above tumours were twelve times more likely to be detected across readers (sensitivity 0.88). Variability was therefore concentrated in anterior lesions and low-grade disease. This is on the lower end of the agreement range for mpMRI (κ = 0.34–0.70 depending on zone, category, and reader experience) [8]. mpMRI advantages include allowing asynchronous reviews by multiple radiologists, structured reporting, reader experience, and less variability with lesion location.

Structured training reduces this variability. Since 2018, Exact Imaging introduced a four-stage progressive feedback program requiring novice microUS operators to meet nine biopsy quality metrics: image quality, lesion identification, target accuracy, systematic sampling, core adequacy, procedural efficiency, patient safety, documentation, and pathologic correlation. Retrospective evaluation of this program found a 17% absolute increase in csPCa detection after formal training [48]. Inter-reader variability should improve with further structured training programs, quality assurance and benchmarking, AI assistance, and updated PRI-MUS criteria.

## 7. Active Surveillance and Restaging Biopsy

Active surveillance (AS) is the SOC for grade group 1 and select grade group 2 prostate cancers. Imaging during AS must detect subtle changes in visible lesions. microUS plays a role here in detecting changes in PRI-MUS.

Several studies have compared microUS and mpMRI in AS populations [49]. In a study of 128 patients with grade group 1 disease on AS undergoing confirmatory biopsy, microUS and mpMRI did not differ significantly in the detection of grade group 2 or above disease. Importantly, there was no upgrading of disease when both microUS and mpMRI were negative [50]. A separate two-centre retrospective comparison of microUS and mpMRI targeted biopsy of 236 men on AS between 2017 and 2022 found that microUS detected significantly more grade group ≥ 2 cancers (27% vs. 16%, *p* = 0.033) and within target lesions (24% vs. 12%, *p* = 0.033) [51]. A total of 24 men (20%) had a negative mpMRI but a suspicious microUS, and 4% of them had csPCa. No patient with a negative microUS had csPCa. For all cancers, microUS demonstrated a sensitivity of 93% and an NPV of 87%, with lower specificity (46%) and PPV (61%). The pattern of high sensitivity and modest specificity exists in the AS population. The Micro-Ultrasound in Cancer—Active Surveillance (MUSIC-AS) trial is an active prospective trial evaluating whether microUS is non-inferior to mpMRI for men on AS [19].

## 8. Local Staging and Tumour Characterisation

Accurate local staging influences surgical and radiotherapy planning, and current guidelines recommend an mpMRI for pre-operative local staging. Extra-prostatic extension (EPE), such as extracapsular extension or seminal vesicle invasion, can be seen on imaging if not already confirmed on pathology.

MicroUS accurately visualises features of local extension. These include capsular breach, capsular irregularity or bulging, obliteration of the prostatic–seminal vesicle angle, and a hypoechoic pericapsular halo. A feasibility study of 54 men undergoing microUS prior to radical prostatectomy found that capsular bulge and hypoechoic halo were significantly more present in non-organ-confined disease (*p* ≤ 0.016) [52]. A subsequent larger prospective single-institutional study reviewed 140 patients planned for robot-assisted radical prostatectomy. microUS evidence of capsular bulging, breach of prostatic capsule, presence of hypoechoic halo and obliteration of the prostatic–seminal vesicle angle were associated with non-organ-confined disease (*p* < 0.001). The rate of non-organ-confined disease increased from 44% in patients with only one predictor (OR 7.71) to 92% in those with all four predictors (OR 72.00) [53]. The overall NPV was 81%, with a PPV of 83% and an AUC of 0.80, for predicting EPE. Frego et al. developed a microUS-based nomogram to predict EPE and guide nerve-sparing surgery. A total of 295 patients underwent microUS the day prior to their robot-assisted radical prostatectomy. microUS correctly identified EPE in 84 of 122 patients who had EPE, with a sensitivity and a specificity of 69% and 84% and an AUC of 0.77 [54]. The complete nomogram incorporating pre-operative variables achieved a predictive accuracy of 0.86. External validation and a head-to-head comparison of microUS against mpMRI for local staging is an unmet need.

The data on microUS to characterise disease phenotype is evolving. Radiogenomic analysis has shown that mpMRI visibility largely overlaps with the driving factors for tumour aggressiveness and mpMRI invisible tumours [55]. The work by Corona et al. suggests that microUS visibility and echogenicity may carry similar prognostic information [27].

## 9. Post-Treatment Surveillance and Local Recurrence

Biochemical recurrence following primary treatment occurs in 20–50% of men [56]. Localisation of the prostatic bed or local recurrences guides salvage treatment options. Following radical prostatectomy, conventional 6–12 MHz TRUS has limited resolution for adequate biopsy of small recurrences. mpMRI provides optimal performance for evaluating the prostatectomy bed. PSMA PET outperforms MRI for nodal and distant metastatic recurrence [57].

Kaufmann et al. conducted a single-institution retrospective study of local prostatic fossa recurrence post-radical prostatectomy. They identified 24 patients between 2013 and 2024, of whom 10 underwent microUS-guided transrectal biopsy, and 14 underwent conventional TRUS-guided biopsy. Despite a smaller median lesion size in the microUS group (0.9 cm compared to 2.5 cm), the sensitivity was 89% compared to 43%. No specificity was reported, as all patients were positive for recurrence. Despite the small sample size, potential for selection bias and single operator, the magnitude of difference justifies future prospective analysis of these patients [20].

## 10. Artificial Intelligence and Micro-Ultrasound

Real-time interpretation is a key distinguishing feature of microUS and an important limitation. There is a substantial advantage to real-time diagnosis and targeted biopsy in a single procedure. However, this limits the opportunity for re-review of images, second opinions or systematic auditing at scale. AI-assisted microUS may reduce inter-reader variability and support the learning curve.

### 10.1. Enabling Models: Segmentation and Image Alignment

Most AI work on microUS to date is focused on infrastructure, rather than a usable bedside tool. Deep learning segmentation of microUS prostate images and accurate alignment to a reference standard is the foundational step for any downstream AI model. Several segmentation models address the challenge of distinguishing between prostate and surrounding tissue on microUS. ‘MicroSegNet’ was trained on 55 patient images and then subsequently validated on an independent 20-patient set. It achieved a Dice coefficient of 0.94 for prostate capsule segmentation, outperforming conventional and expert reviewers [58]. Hung et al. developed a user-guided 3D segmentation model that predicts whole prostate volume after the user places a few points on boundary slices. It was trained on 55 patients and tested on 758 images from 20 patients. The performance was similar between a clinical expert and a non-expert [59]. Al-Qurri et al. proposed a dual-encoder segmentation model that achieved a Dice coefficient of 0.94 on its micro-ultrasound datasets [60]. On the alignment side, a deep learning registration of in vivo microUS to whole-mount histopathology reached an error of approximately 2.8 mm [61]. Methods now exist to format non-uniform microUS sweeps into standard planes that are comparable to mpMRI and histopathology [62].

### 10.2. Cancer Detection and Grading

Models detecting cancers have been tested against human experts. Initial models demonstrated that microUS texture contains a detectable cancer signal for AI models. Two of these, TRUSformer and TRUSworthy, achieved AUC values around 0.80–0.81 for csPCa detection [63,64]. The more recent ProstNFound family of models generates a cancer likelihood map and a patient-level risk score. In a multicentre cohort of 693 patients, it reached a sensitivity of 90% and a specificity of 40%, comparable to human readers for microUS [65]. Its successor model (ProstNFound+) was validated prospectively at a new site on data acquired after five years of training, with no loss of performance against PRI-MUS [65].

Another model, ProMUS-NET, has had the most compelling performance. It was trained to localise grade group 2 and above cancers, achieving an AUC of 0.92. It detected more csPCa than six expert urologists (lesion-level sensitivity 73% vs. 58%; patient-level 77% vs. 66%) [66].

Imran et al. recently reported the first dedicated AI-screening microUS study. Their model analysed 145 men undergoing microUS-guided biopsy and reached an AUC of 0.87, outperforming both PSA and DRE-based assessment, for csPCa [21].

### 10.3. Reader Support and the Learning Curve

AI models may shorten the learning curve and aid novice microUS users. The DeepTRUS model provides a heatmap overlay to aid performance in microUS sweeps. The developers trained DeepTRUS on 100 microUS sweeps with 50 csPCa and 50 benign prostates. A small pilot study was conducted with five novices. On its own, DeepTRUS has modest performance (AUC 0.69). Despite this, the heatmaps generated improved the microUS interpretation of the five novices. The AUC improved from 0.46 to 0.56 with overlay, whilst the sensitivity improved from 60–72% to 80–88% with DeepTRUS. AI assistance improved AUC and sensitivity at every stage [67].

### 10.4. Current Status and Clinical Adoption

Cella et al. conducted a systematic review of the literature to December 2025, identifying studies across detection, segmentation and registration. These demonstrate promising early results, with accuracy approaching or matching expert readers [68].

The aforementioned models that assess anatomy (i.e., the MicroSegNet, the Hung model and the Al-Qurri model) have only been internally validated and have no clinical use currently [58,59,60]. Similarly, the TRUSformer, TRUSWorthy and ProMUS-NET models for cancer detection are purely experimental [63,64,66]. ProstNFound+ remains the only prospective validation study.

No microUS AI model has received clearance for clinical use, and none are commercially available within the existing ExactVu MicroUS platform. All published models remain investigational. The barriers to wider or routine clinical adoption are external validation across multiple sites and users to ensure reproducibility, clinical endpoints and ultimately regulatory clearance.

## 11. Workflow, Cost, and Environmental Considerations

microUS in the prostate cancer diagnostic paradigm has workflow advantages. It combines imaging and biopsy into one room, one procedure and one proceduralist. It removes the additional separate scan appointment, the radiology reporting queue, the second outpatient visit for imaging review and biopsy planning and the mpMRI/TRUS fusion step if software is to be used. The biopsy schedule is a significant source of delay, accounting for up to 70% of delays in a UK series within an efficient mpMRI-first pathway [69]. In Canada, the single-encounter pathway shortens time to diagnosis and reduces patient anxiety and treatment delay [16]. There is limited health–economic evidence for microUS use to date, though cost-effectiveness modelling has favoured mpMRI pathways over TRUS-only pathways. However, the CADMUS investigators noted that the microUS device cost is approximately one-tenth to one-twentieth of the modern MRI, which is particularly advantageous for low-income and limited resource settings [11].

Similarly, there is limited evidence regarding the environmental impact. Life-cycle reports suggest an approximate 35-fold difference in CO_2_ emissions, 6.2–76.2 kg for MRI vs. 0.1–1.2 kg for conventional US. Annual equipment-level emissions for an MRI are 53 metric tonnes, whilst for a US, they are 0.3 metric tonnes [70]. No life-cycle assessment, energy consumption measurement, or emission audit specific to microUS has been published. An inference can be made due to shared hardware characteristics, such as no requirement for shielded rooms, no cooling requirements, and a portable cart from a standard mains supply. Any environmental advantage attributed to microUS is provisional until platform-specific data are generated.

Beatrici et al. explored microUS as a gatekeeper and provided data regarding resource optimisation by using microUS as a front-line tool for prostate diagnostics. They enrolled 425 men with clinical concern for prostate cancer due to PSA elevation, and all underwent a microUS as the first step. Overall, 53% (224/425) of the men had a negative microUS and were triaged away from further invasive work-up. Of these, 92% did not undergo subsequent mpMRI, and 86% avoided biopsy altogether. Median follow-up was 39 months with close surveillance. These men subsequently had significantly lower all prostate cancer and csPCa detections (HR of 0.12 and 0.09, respectively). By avoiding the conventional pathway, 207 mpMRI were avoided and 192 biopsies were avoided. Only two patients (1% of all negative microUS patients) were ultimately found to have csPCa [71].

The OPTIMUM trial authors argue that environmental differential should be considered alongside diagnostic performance and cost to determine the optimum prostate cancer diagnostic pathway [15].

## 12. Limitations of the Current Evidence

There are several limitations of the current evidence. Firstly, if considering microUS as a competitor to mpMRI, the OPTIMUM trial remains the sole randomised comparative evidence for microUS compared to mpMRI [15]. The other comparative studies have typically not blinded the microUS operator to prior mpMRI.

The patient population studies are heterogeneous. MicroUS has been applied in biopsy-naïve men, patients with negative biopsy, equivocal patients with PI-RADS 3 lesions and AS. The biopsy approach has also varied in the literature, as well as the comparative mpMRI modality (cognitive vs. fusion).

The specificity of microUS remains relatively low across multiple meta-analyses, with pooled estimates ranging from 0.25 to 0.41 depending on the comparator and reference standard used [17,39]. This is the principal diagnostic limitation of the technology. Low specificity means that a proportion of PRI-MUS ≥ 3 lesions are false positives, and a microUS-first pathway will therefore direct some men to biopsy who do not have csPCa, with the associated risks of infection, bleeding, discomfort, and anxiety. This must be weighed against the high negative predictive value of microUS, which supports its use as a rule-out rather than a rule-in test, particularly when combined with PSA density [71]. More recent data from the OPTIMUM trial and subsequent studies suggest that specificity improves with operator experience, although the magnitude of improvement and the volume of experience required have not been quantified [15].

Operator and inter-reader heterogeneity is a substantial barrier, though training programs and AI are pathways to address this. These difficulties are heightened by anterior gland tumours and lower-grade disease.

As it stands, there is also a paucity of cost-effectiveness models and head-to-head comparisons for local staging, post-treatment recurrence and AS. The research priorities in microUS also include refinement of PRI-MUS to incorporate echogenicity and anterior zone scoring, as well as standardisation of training, accreditation and quality assurance.

All clinical microUS evidence to date derives from the single commercially available platform. No vendor-agnostic validation exists; therefore, the generalizability of published performance to any future high-frequency platforms is unknown.

The published micro-ultrasound evidence derives predominantly from high-income health systems in North America and Europe; prospective data from Asian, African, and broader South American populations are lacking, and validation in these settings—where prostate cancer epidemiology, ethnic diversity, and health system infrastructure differ—remains an important priority for future research.

## 13. Ongoing and Future Trials

There are several ongoing prospective trials across active surveillance, screening, focal therapy and novel surgical techniques as of June 2026 (Table 2). Two are evaluating microUS in AS (MUSIC-AS and MUS-AS)—one as a paired, non-inferiority test at the time of confirmatory biopsy and the other as a tool for mpMRI-invisible patients on AS. MUSIC-Screen is a multicentre phase III non-inferiority trial of biopsy-naïve patients, comparing microUS to mpMRI. Two are in novel therapeutic applications with microUS-guided focal laser ablation of prostate cancers and microUS-guided nerve-sparing during radical prostatectomy.

## 14. Conclusions

microUS has become an established tool in the armamentarium of prostate cancer diagnosis. The OPTIMUM trial provides level 1 evidence demonstrating non-inferiority of microUS to mpMRI for csPCa. Numerous meta-analyses report comparable sensitivity to mpMRI with low rates of insignificant disease detection. Real-world data mirrors the findings in trials, including the use of microUS without software fusion. microUS has potential roles in AS, restaging, and post-prostatectomy local recurrence. Aside from its clinical utility, it potentially poses practical advantages in workflow, cost-effectiveness and environmental burden. The inter-reader variability and a significant learning curve are challenges. AI-enhanced microUS promises to reduce inter-reader variability.

microUS is an appealing primary imaging modality for the evaluation of men with suspected prostate cancer.

## Figures and Tables

**Table 2:** Ongoing microUS clinical trials registered on ClinicalTrials.gov.

Trial (NCT)	Location/Sponsor	Design and Setting	*n*	Status (Start → Primary Completion)	Primary Endpoint
MUSIC-AS (NCT05558241)	Multicentre: Canada, USA, Italy; University of Alberta	Prospective, paired, non-inferiority; AS confirmatory biopsy	210	Recruiting (November 2022 → August 2026)	Non-inferiority of microUS for GG ≥ 2 detection at confirmatory biopsy
MUS-AS (NCT07386171)	Montreal, Canada; McGill University Health Centre	Single-arm, two-stage phase II diagnostic; AS or new diagnosis with negative/stable mpMRI	90	Not yet recruiting (March 2026 → March 2027)	Sensitivity and specificity of microUS for csPCa (GG ≥ 2)
MUSIC-Screen (NCT06626022)	Multicentre: Canada, USA, France, Norway, Spain; University of Alberta	Phase III, randomised, non-inferiority; biopsy-naïve screening	1284	Recruiting (May 2025 → November 2029)	Non-inferiority of microUS to mpMRI for GG ≥ 2 detection
microUSgFLA (NCT07339943)	Toronto, Canada; University Health Network (collab. Exact Imaging, CLS AB)	Single-arm safety/effectiveness pilot; focal laser ablation, intermediate risk	7	Recruiting (April 2025 → May 2026)	Procedure-related adverse events (CTCAE v5.0) and technical success
SAFE Nerve-Sparing (NCT06945315)	New York, USA; Icahn School of Medicine at Mount Sinai	Phase III RCT; nerve-sparing RARP ± saline-assisted fascial engorgement	196	Recruiting (November 2023 → July 2027)	Erectile function recovery (SHIM score, to 24 months)

## Data Availability

No new data were created or analyzed in this study. Data sharing is not applicable to this article.

## Abbreviations

The following abbreviations are used in this manuscript:

microUS	micro-ultrasound
csPCa	clinically significant prostate cancer
mpMRI	multiparametric magnetic resonance imaging
PRI-MUS	Prostate Risk Identification using Micro-UltraSound
PI-RADS	Prostate Imaging Reporting and Data System
TRUS	transrectal ultrasonography
AS	active surveillance
RCT	randomised controlled trial
NPV	negative predictive value
PPV	positive predictive value
AUC	area under the curve
ISUP	International Society of Urological Pathology
GG	grade group
RP	radical prostatectomy
EPE	extra-prostatic extension
AI	artificial intelligence
